# Mitotic Checkpoint Kinase Mps1 Has a Role in Normal Physiology which Impacts Clinical Utility

**DOI:** 10.1371/journal.pone.0138616

**Published:** 2015-09-23

**Authors:** Ricardo Martinez, Alessandra Blasina, Jill F. Hallin, Wenyue Hu, Isha Rymer, Jeffery Fan, Robert L. Hoffman, Sean Murphy, Matthew Marx, Gina Yanochko, Dusko Trajkovic, Dac Dinh, Sergei Timofeevski, Zhou Zhu, Peiquing Sun, Patrick B. Lappin, Brion W. Murray

**Affiliations:** 1 Oncology Research Unit, Pfizer Worldwide Research and Development, 10724 Science Center Drive, San Diego, CA, 92121, United States of America; 2 Worldwide Medicinal Chemistry, Pfizer Worldwide Research and Development, 10724 Science Center Drive, San Diego, CA, 92121, United States of America; 3 Drug Safety Research and Development, Pfizer Worldwide Research and Development, 10646 Science Center Drive, San Diego, CA, 92121, United States of America; 4 Department of Cell and Molecular Biology, The Scripps Research Institute, 10550 North Torrey Pines Road, La Jolla, CA, 92037, United States of America; Wayne State University, UNITED STATES

## Abstract

Cell cycle checkpoint intervention is an effective therapeutic strategy for cancer when applied to patients predisposed to respond and the treatment is well-tolerated. A critical cell cycle process that could be targeted is the mitotic checkpoint (spindle assembly checkpoint) which governs the metaphase-to-anaphase transition and insures proper chromosomal segregation. The mitotic checkpoint kinase Mps1 was selected to explore whether enhancement in genomic instability is a viable therapeutic strategy. The basal-a subset of triple-negative breast cancer was chosen as a model system because it has a higher incidence of chromosomal instability and Mps1 expression is up-regulated. Depletion of Mps1 reduces tumor cell viability relative to normal cells. Highly selective, extremely potent Mps1 kinase inhibitors were created to investigate the roles of Mps1 catalytic activity in tumor cells and normal physiology (PF-7006, PF-3837; *K*_i_<0.5 nM; cellular IC_50_ 2–6 nM). Treatment of tumor cells *in vitro* with PF-7006 modulates expected Mps1-dependent biology as demonstrated by molecular and phenotypic measures (reduced pHH3-Ser_10_ levels, shorter duration of mitosis, micro-nucleation, and apoptosis). Tumor-bearing mice treated with PF-7006 exhibit tumor growth inhibition concomitant with pharmacodynamic modulation of a downstream biomarker (pHH3-Ser_10_). Unfortunately, efficacy only occurs at drug exposures that cause dose-limiting body weight loss, gastrointestinal toxicities, and neutropenia. Mps1 inhibitor toxicities may be mitigated by inducing G_1_ cell cycle arrest in Rb1-competent cells with the cyclin-dependent kinase-4/6 inhibitor palbociclib. Using an isogenic cellular model system, PF-7006 is shown to be selectively cytotoxic to Rb1-deficient cells relative to Rb1-competent cells (also a measure of kinase selectivity). Human bone marrow cells pretreated with palbociclib have decreased PF-7006-dependent apoptosis relative to cells without palbociclib pretreatment. Collectively, this study raises a concern that single agent therapies inhibiting Mps1 will not be well-tolerated clinically but may be when combined with a selective CDK4/6 drug.

## Introduction

Error-free chromosome segregation during the cell cycle is essential to the viability of normal cells. This process occurs during the metaphase-to-anaphase transition and is enforced through a complex circuitry of proteins known as the mitotic checkpoint (spindle assembly checkpoint, SAC) [1]. The transition from metaphase to anaphase is inhibited by the mitotic checkpoint until sister chromatid are attached to microtubules of the mitotic spindles and have established the correct bipolar microtubule-kinetochore attachments [2–4]. High fidelity of chromosomal separation requires that each sister kinetochore is attached to a single pole. The mitotic checkpoint is sufficiently sensitive to prevent anaphase when even only one chromosome is not correctly attached [3, 5, 6]. Failure of the mitotic checkpoint can lead to chromosomal imbalances (aneuploidy) arising from uneven sister chromosomal segregation during cell division which results in a phenotype called chromosomal instability (CIN) [7]. Mechanical outcomes of this dysregulation included altered kinetochore attachment, incorrect spindle tension, and improper chromosomal alignment. Core proteins of this process are known (MAD1/2/3, Bub1/3, BubR1, Mps1) [5, 6]. Mps1 enzymatic activity has been proposed to have a key role in the sensitivity of the mitotic checkpoint by enabling cells to resolve merotelic attachments [8]. In addition, the core proteins are regulated to increase the fidelity of the checkpoint. For example, polo-like kinase 1 enhances Mps1 activity and localize it to the mitotic checkpoint [9]. Tumor cells are different because they readily tolerate genomic instability. Further, tumors are thought to require a certain level of CIN to maintain viability [10]. Relaxation of mitotic checkpoint processes may be a mechanism to diversify the genotypic landscape by enabling genomic and mutational plasticity (mutator phenotype) [11, 12]. Genomic instability may be linked to the evolution of drug resistance [13]. Given the critical role of aneuploidy in cancer, CIN may be a process to target therapeutically.

One hypothesis on how to exploit the mitotic checkpoint process is based on the concept that genomic instability comes at the expense of compromised viability (highly optimized tolerance [14]). Therefore, pharmacological augmentation of genomic instability may be selectively detrimental to tumor cells. To test this hypothesis, we investigated the core mitotic checkpoint protein monopolar kinase 1 (Mps1, TTK) because of studies implicating this serine/threonine kinase in mitotic checkpoint dysregulation. Mps1 is a component of the CIN expression signature and a highly expressed gene in multiple cancer types including breast cancer [15]. Mps1 expression correlates with poor prognosis in breast cancer [16] as well as other tumor types [17]. Elegant *in vitro* and *in vivo* studies of Mps1 depletion have shown a requirement for a high level of Mps1 in tumor cells [10]. When Mps1 is reduced 50–60% by inducible shRNA, breast cancer xenograft tumor growth is decreased [10]. It should be noted that the approach is complicated by a concomitant reduction of mitotic checkpoint protein BubR1 as well as elimination of noncatalytic Mps1 functions critical to the mitotic checkpoint [18, 19]. The catalytic activity of Mps1 is known to be involved in the regulation of the mitotic checkpoint although the molecular underpinnings are still to be fully defined [20]. Taken together, these studies implicate Mps1 in facilitating a aneuploidy-tolerant state in tumor cells [10] and support Mps1 inhibition as a promising monotherapy [21].

Although shRNA knockdown studies have shown a requirement for a high level of Mps1 *in vitro* and *in vivo* [10], their limitations necessitate a complementary approach to gain a deeper understanding of the role that Mps1 catalytic activity plays in the mitotic checkpoint. Small molecule inhibitors with sufficient biological and pharmacological properties can address this gap by selectively and completely blocking Mps1 catalytic activity. To date, many Mps1 inhibitors have been reported [17, 22–30]. Some are studied only *in vitro* [17, 25, 26, 30] while others have analysis extended *in vivo* [22, 23, 25, 27–29]. Chemical biology analysis has limitations because inhibitors can have suboptimal properties which complicate the interpretation of experimental results (e.g. not specific enough, insufficient potency, or incapable of sustained inhibition). Further, dosing schedule and route of delivery have critical roles in the resulting pharmacology–intravenous twice weekly dosing will be very different than daily oral dosing. These complexities may be at the root of apparently contradictory Mps1 inhibitor findings. Some studies report that Mps1 inhibition is well-tolerated [22, 27, 28] while others report the opposite [25, 29]. To date, no study has reported on the pathology of Mps1-targeted therapies. Even though other mitotic checkpoint targeted therapies have achieved complete tumor regressions (e.g. CENP-E inhibitors [31]), no Mps1 inhibitor has produced profound efficacy in the absence of confounding toxicities. This can be due to either insufficient pharmacokinetic properties of the inhibitors or because complete Mps1 inhibition is not well-tolerated. In the clinic, tumors must regress for an anti-mitotic therapy to achieve a partial or complete response. In order for Mps1 to be considered a viable chemotherapy target, significant efficacy needs to occur at well-tolerated doses.

In the presence work, highly-selective Mps1 kinase inhibitors are created to study the role of the catalytic function of Mps1 in tumor cell survival. Mps1 inhibitors reported in the current study are among the most potent inhibitors described to date (*K*_i_<0.5 nM, cellular IC_50_<6 nM) with sufficient pharmacokinetic properties to evaluate the consequences of sustained Mps1 inhibition *in vivo*. These studies reveal that effects on tumor cellular viability occurs concomitant with mechanism-based, dose-limiting toxicities. *In vitro* studies show that Mps1 inhibitor cytotoxicities are ameliorated when combined with a cytostatic, selective CDK4/6 drug that pauses the cell cycle progression of normal cells while keeping tumor cells vulnerable to Mps1-mediated cell death. Taken together, these studies refine the therapeutic utility of targeting Mps1 and build support for using highly-selective CDK4/6 drugs as chemo-protective agents.

## Materials and Methods

### Cell lines

The tested cell lines used to evaluate the activity of PF-7006 were obtained from American Type Culture Collection (ATCC, Manassas, VA). Cell cultures were expanded by serial passaging and stocks frozen after 3–5 passages.

### Chemistry

PF-7006 and PF-3837 (**S1 Fig**) were synthesized at Pfizer, La Jolla.

### Mps1 knockdown studies

Human Mps1 siRNA (LU-004105-00-005) and non-silencing siRNA sequence (0-001206-13-20) were purchased from Dharmacon and cells were transfected by using LipofectAMINE plus (Life Technologies, Carlsbad, CA) following the manufacturer’s instructions. The siRNA target the following regions of Mps1 (NM-003318): 897–915, 1441–1459, 1821–1832, and 2036–2054. Human Mps1 shRNAmir (GACAGATGATTCAGTTGTA) (Kwiatkowski et al., 2010) and non-targeting shRNA (CAACAAGATGAAGAGCACCAA (derived from SHC002, Sigma) sequences were introduced into a TRPZI lentiviral expression vector (CELLECTA). 293T cells were cotransfected with a lentiviral packaging system and the on-target or non-targeting lentiviral DNA. After culturing for 3 days, media was harvested and viral titer determined. Lentiviral infection and stable clone production was performed on BT549 cells seeded in 6 well plates and grown to 80% confluence. On-target or non-targeting lentivirus (1 mL) was added to cells. Puromycin was added starting at a concentration of 0.2 μg/mL and increased to 2 μg/mL until resistant cells expanded.

### Expression and purification of Mps1 protein

Activated Mps1 enzyme was prepared as follows. Mps1 catalytic domain (aa 510–857) containing N-terminal sequence MKHHHHHHDYGILTTENLYFQGSA was expressed in E.coli and purified by affinity chromatography following treatment with TEV protease (to remove polyHis tag) and Lambda phosphatase (to completely dephosphorylate protein). The enzyme was activated by autophosphorylation with MgATP at 0°C for 15 min to introduce a single phosphate (activation loop Thr_676_), as verified my mass-spectrometry, and MgATP was removed by buffer exchange.

### Biochemical assay

Mps1 inhibition was measured in a mobility shift assay format that electrophoretically separates the fluorescently labeled peptide substrate and phosphorylated product following the kinase reaction. The reaction mixture was applied to a LabChip3000 system (Caliper Life Science, Hopkinton, MA), and the product /substrate peptide peaks were separated. The kinase reaction was quantitated by the product ratio calculated from peak heights of product (P) and substrate (S) peptides (P/(P+S)). Dose-response analysis was performed were tested with a mobility-shift assay that combines the basic principles of capillary electrophoresis in a micro-fluidic environment. The 60 minute enzymatic reaction contained 5 nM Mps1 catalytic domain (aa 510–857), 60 μM (~K_m_-level) ATP, 5 μM phosphoacceptor peptide substrate (5FAM-RQRRSIDDTIDSTRLFE-NH_2_; CPC Scientific, Sunnyvale, CA), 10 mM MgCl_2_, 4% DMSO (with or without inhibitor), 1 mM DTT, 0.01% Tween-20, 50 mM HEPES (pH 7.5). The *K*_i_ values were determined from 11 inhibitor concentrations with a 1:3 serial dilution starting from 3 μM. Reaction rates were corrected for a background reaction in the presence of an excess of EDTA (15 mM). Corrected rates were fit to a tight-binding (quadratic) equation for competitive inhibition (Morrison, 1969). PF-7006 and PF-3837 were screened at 1 μM toward a panel of protein kinases using Millipore KinaseProfiler screening service uses a radiometric kinase assay to assess inhibitor potency at a *K*_m_ concentration of ATP (Gao et al., 2013). In addition, inhibitors profiled against protein kinases by Carna Biosciences (Kobe, Japan) with the mobility-shift assay format Caliper LabChip3000. Typical reactions were 20 μL, contained 120 ng/mL protein kinase, *K*_m_-level of ATP, 1.0 μM peptide substrate, 5 mM MgCl_2_, 2 mM DTT, 0.01% Triton X-100, 6.25% DMSO in 20 mM HEPES pH 7.5. The mixture was incubated in a 384-well polypropylene plate at room temperature for an hour and terminated by the addition of 60 μL of QuickScout Screening Assist MSA Buffer (Carna Biosciences, Kobe, Japan). Reactions were run for between 1 and 5 hours dependent on the individual kinase and quantified similarly to the Mps1 assays.

### Cellular ELISA assay for phosphorylation of Histone H3-Ser_10_

MDA-MB-468 cells were seeded in a 96-well plate in RPMI1640 media supplemented with 10% fetal bovine serum (FBS) and 1% penicillin-streptomycin at 37°C with 5% CO_2_ until 80–90% confluence. The cells were treated with a CENP-E inhibitor PF-2771 (Kung et al., 2014) at 75 nM for 16 hours then added Mps1 inhibitors for addition 2 hours. Results in their release from cell cycle arrest and ultimately into mitosis which is then quantified by measuring the expression of Phospho-Histone H3. Phospho-HH3-Ser_10_ was measured in triplicate using PathScan phospho-HH3-Ser_10_ sandwich ELISA kit (Cell Signaling Technology). Data were exported and analyzed using a four-parameter IC_50_ (GraphPad Prism).

### Cell proliferation assays

PF-7006 or PF-3837 were added to cells seeded in 96-well plates. The number of cells seeded (1,000–3,000) depended on growth characteristics of each cell type and normalized proliferation rates. Cells were seeded in a 96-well plate in RPMI1640 media supplemented with 10% fetal bovine serum (FBS) and 1% penicillin-streptomycin at 37°C with 5% CO_2_ until 80–90% confluence. Ten different concentrations of compound used were based on a half-log increment between 1 nM and either 1 μM or 25 μM. Cells were pre-incubated at 37°C for 7 days before assessing viability with the Cell Titer Glow (CTG) reagent (Promega). Untreated control cells were 80–90% confluent after seven days of culture. Data were fitted into a sigmoidal curve-fitting program to calculate IC_50_ values.

### Flow cytometry

Analysis of cell cycle distribution was performed by flow cytometry. These experiments were done independently at least 3 times and produced similar results. Cells were grown in RPMI1640 media supplemented with 10% fetal bovine serum (FBS) and 1% penicillin-streptomycin at 37°C with 5% CO_2_. A MDA-MB-468 cell culture containing 1 to 2 x 10^6^ cells was treated with the inhibitor (25 or 50 nM PF-7006 or PF-3837) for 96 h. The cells were fixed in 70% ethanol and stained with propidium iodide using the CycleTEST Plus kit (Becton Dickinson). The samples were analyzed on a Becton Dickinson FacsCaliber Instrument. The proportion of cells in G1, S, G2-M, aneuploidy or non-gated (sub-G1) was determined using Cell Quest v.2.0 software (Becton Dickinson). Gates were set based on control cells and used for all of the subsequent sample treatments. For Mps1 treated cells, there is cell death/debris to account for, outside of the subG1 fraction which is picked up with propidium iodide staining. When the cell populations were gated for the experiment, the cellular debris that did not stain well was excluded.

### Analysis of cell cycle proteins in tumor cells

Antibodies used are as follows: Mps1 (Pierce, cat. MA1-24959), phospho-Mps1 (pT33/pS37) (Life Technologies, cat. 44-1325G), cyclin B1 (Cell Signaling Technology, cat. 4135; aurora-B/AIM1 (Cell Signaling Technology, cat. 3094), phospho-aurora-A(Thr288)/-B(Thr232)/-C(Thr198) (Cell Signaling Technology, cat. 2914, 1:500 dilution), securin (Abcam, cat. ab3305-500), phospho-HH3 (Ser_10_) (Cell Signaling Technology, cat. 9701), survivin (Cell Signaling Technology, cat. 2808), anti-BubR1 (Bethyl Laboratories), borealin (Cell Signaling Technology, cat. sc-130705) anti-phosphohistone-H2AX Ser_139_ (Millipore; Billerica, MA), Actin (Santa Cruz Biotechnology). Cells were seeded in a 96-well plate in RPMI1640 media supplemented with 10% fetal bovine serum (FBS) and 1% penicillin-streptomycin at 37°C with 5% CO_2_ until 80–90% confluence. Studies were performed in asynchronous or in cells arrested in G/M by pre-treatment with docetaxel, an agent known to induce mitotic arrest. Asynchronous cells were treated with the inhibitor at increasing concentrations for 72 hours, or at 100 nM for different lengths of time, up to 72 hours. For studies performed on synchronized cells, docetaxel at 100 nM was added to cells 16 hours prior incubation with the Mps1 inhibitor (PF-7006, PF-3837). Cells were incubated in the presence of PF-7006 for 4 hours prior to harvesting. The cell lysates were prepared by scraping the cells in the presence of cold PBS buffer containing protease and phosphatase inhibitors. Proteins were transferred to nitrocellulose membranes and incubated overnight 4°C with the primary antibodies. HRP-conjugated secondary antibodies (GE Healthcare) were incubated (2h, RT), detected using a SuperSignal West Dura chemiluminescent substrate (Pierce SuperSignal West Dura; Thermo Fisher Scientific, Rockford, IL), and imaged (AlphaInnotech imager; Cell Biosciences, Santa Clara, CA).

### *In vivo* tumor model studies

All animal experimental procedures complied with the Guide for the Care and Use of Laboratory Animals (Institute for Laboratory Animal Research, 1996) and were approved by the Pfizer Global Research and Development-La Jolla Institutional Animal Care and Use Committee. Animals were given a minimal of 3 days to acclimatize before tumor cell implantation. Animals under study were monitored daily. Animals were manually restrained for <1 minutes during tumor measurement and dosing. Animals in distress were put into separate cages with extra soft bedding and fed on liquid gel food. Animals were euthanized when any of the following was observed: 20% body weight loss; tumor size reaches 1500 mm^3^; tumors with severe necrosis; shallow breathing; lethargy, hunched back, rough hair coat, severe dehydration or moribund status. Method of euthanasia used in this study is cervical dislocation under isoflurane anesthesia or CO_2_ overdose. HCC1806 tumor cells (3x106) cells were implanted in the mammary fat pad of CB17/lcr.Cg-Prkdc^scid^Lyst^bg^ female mice (Charles River Breeding Laboratories). For in vivo dosing, PF-7006 formulation consisted of a wet-milled crystalline nanosuspension of the compounds suspended in 2.5% polyvinyl pyrrolidone, 0.5% Solutol HS 15 in water. The formulations were prepared by milling a suspension of the compounds using yttrium coated zirconia beads (500 μm diameter) at 1000 rpm on a super magnetic stirrer for at least 6 hours. The particle size of active following the milling process was typically determined to be less than 300 nm (number weighted d90). For efficacy studies, when HCC1806 xenograft tumors reached 150–200 mm3, mice were randomly distributed into treatment groups of 12, such that the mean value of tumor size was identical for all groups. PF-7006 was administered orally (PO) to groups of 12 mice at 0.2 mg/kg QDx14, 1 mg/kg QDx14, 5 mg/kg Q2Dx7, and 25 mg/kg Q3Dx7. Tumor volumes were recorded by calipers three times per week with the final measurement taken three days after the last dose. Tumor growth inhibition (TGI) was calculated using the formula 100 × (1-ΔT/ΔC), where ΔT (treated) and ΔC (control) are the mean tumor volume changes between one day after the last dose and the first day treatment. Time-to-progression endpoint and associated tumor growth delay determinations was calculated using median days to reach to two doublings of initial tumor size. Statistical comparisons among animal treatment groups were made using one-way ANOVA with Dunnett’s post-tests. Body weight was recorded throughout the study as a coarse measure of drug toxicities. Animals were euthanized when tumors exceeded a volume of ~1,500 mm^3^ or if the physical condition of the animal warranted intervention.

### Pharmacokinetic and pharmacodynamic studies

Pharmacokinetic/pharmacodynamic (PK/PD) analysis were performed in this study using the pHH3-Ser_10_ as the pharmacodynamic marker measured by ELISA. Mice bearing orthotopically implanted HCC1806 xenograft tumors (300–500 mm^3^) were evaluated for plasma drug concentrations and tumor levels of phosphohistone H3-Ser_10_. Docetaxel at 30 mg/kg (single dose) was administered intra-peritoneally 24 hours prior the Mps1 inhibitor. PF-7006 was administered orally at 4 different doses, ranging from 0.2 mg/kg to 25 mg/kg. Tumor and blood were collected at different times post dose (3 mice/time point, error bars are standard deviation). For pharmacodynamic analysis, tumors were excised and cut into two pieces, one snap frozen in liquid nitrogen, and the other fixed in 10% neutral-buffered formalin. Frozen tumor tissue was ground to fine powder, and homogenized in ice-cold lysis buffer (Cell Signaling Technologies), supplemented with protease and phosphatase inhibitors. Following protein concentration determination, tumor lysates were subjected to ELISA assay: lysates were plated in triplicate on phospho-Histone H3 (Ser10) coated ELISA plates (Cell Signaling Technologies), as described by manufacturer (error bars are standard deviation). For the immunohistochemistry studies, formalin-fixed tumors sections were stained with an antibody for phospho-Histone H3 (Cell Signaling Technologies, #9701), as described by manufacturer. For pharmacokinetic analyses, plasma was collected in heparinized vacutainer tubes following intra-cardiac puncture. Drugs were extracted from plasma using three volumes of acetonitrile containing internal standards. Drug concentrations were determined using reverse-phase high performance liquid chromatography and mass spectrometry analysis.

### Pathology

Selected tissues were chosen for the evaluation of toxicity from a single maximum tolerated dose (MTD) study that had variable doses and schedules (n = 3): daily (vehicle, 0.2, 1 and 5 mg/kg PF-7006), every 2 days for 2 weeks (vehicle, 0.2, 1 and 5 mg/kg PF-7006), or 25 mg/kg PF-7006 every 3 days for 14 days. All animals at the 5 mg/kg daily dose and a single animal at the 25 mg/kg q2-3 days were euthanized moribund prior to the scheduled study termination. At necropsy, representative sections of stomach, small and large intestine, and bone marrow (sternum) were collected and immersed fixed for 24–48 hours in 10% neutral buffered formalin (NBF). Sternum samples were decalcified in EDTA. All tissues were subsequently processed to 4 μm thick tissue sections for hematoxylin and eosin staining. All slides were reviewed by a board certified veterinary pathologist, described morphologically and scored subjectively. All evaluated tissues were evaluated for soft tissue degeneration/necrosis, apoptosis, mitotic activity and mitotic morphology, using WNL (within normal limits), minimal, mild, moderate, or marked as indicators of severity.

### Protective effects of CDK4/6 inhibition against apoptosis induced by Mps-1 inhibitor in human bone marrow mononuclear cells

Human bone marrow mononuclear cells were purchased from Lonza (Walkersville, MD). The cells were cultured in the hematopoietic progenitor growth (HPGM) media (Lonza) supplemented with 10% fetal bovine serum (FBS) and in the presence of the following cytokines (R&D systems, Minneapolis, MN): 25 ng/ml stem cell factor (SCF), 3 U/ml erythropoietin (EPO), 10 ng/ml granulocyte colony-stimulating factor (G-CSF), 10 ng/ml granulocyte-macrophage colony-stimulating factor (GM-CSF), 15 ng/ml thrombopoietin (TPO), 10 ng/ml interleukin 3 (IL-3), 10 ng/ml interleukin 6 (IL-6), and 25 ng/ml Flt3 ligand, in a 37°C 5% CO_2_ and 98% humidity incubator. Apoptosis was measured in triplicate using the Caspase-Glo® 3/7 activation assay (Promega, Madison, WI), according to the manufacturers’ protocols.

### Protective effects of CDK4/6 inhibition against apoptosis induced by Mps-1 inhibitor in rat gastrointestinal cells

IEC-6 small intestine epithelial rat cells (ATCC; CRL- 1592) were maintained in DMEM supplemented with 10% fetal bovine serum, insulin-transferrin-selenium-A (Life Technologies) and pen/strep (Life Technologies, Inc). Cells were used between passage 6 and 16. For cell viability and caspase 3/7 assays IEC-6 cells were plated at a density of 3000–5000 cells per well in white-walled, clear bottom 96-well plates (Costar). The following day, growth media was removed and 100 μL media containing 0.1% FBS and the indicated compounds were added for 24–48 hours. For combination experiments, IEC-6 cells were pre-incubated with 1 μM palbociclib for 24 hours after which the media was removed and fresh media containing the combination of either DMSO or 1 μM palbociclib with the indicated doses of PF-3837 or PF-7006 was added for an additional 24 or 48 hours. Results were confirmed with four experiments each with three replicates per dose (standard deviations used to define error bars). Cell viability was quantified with the CellTiter-Glo® Luminescent Cell Viability Assay (Promega, Madison, WI) according to the manufacturer’s instructions. Caspase 3/7 induction was quantified with the Caspase-Glo® Assay kit (Promega) according to the manufacturer’s instructions. Luminescence was quantified with a Safire2 spectrophotometer (Tecan, Austria).

## Results

### Expression of Mps1 is linked to poor prognosis in breast cancer

Transcriptional analysis of a large cohort of clinically annotated primary fresh frozen breast cancer specimens (1163 breast cancer patients, 160 normal breast tissue samples) (public database, [32]) revealed that many cell cycle genes are significantly elevated in breast cancer (e.g. Mps1, p = 8.32x10^-10^). Mps1 is over-expressed in breast cancer tissue relative to normal-adjacent tissue samples and its expression varies across distinct expression profile segments of breast cancer. The fold change (tumor vs normal adjacent tissue) and statistical significance (two-tailed *t*-test) are as follows: basal-a (4.57-fold, p = 1.34x10^-52^), Her2 (2.81-fold, p = 8.49x10^-27^), luminal B (2.53-fold, p = 2.50x10^-47^), luminal A (1.65-fold, p = 1.39x10^-56^), and basal-b/claudin-low (1.47-fold, p = 2.06x10^-3^). Mps1 expression is prognostic because its up-regulation correlates with significantly shorter disease-specific survival (480 patients, p = 5.53x10^-9^ from log-rank test) (**S2 Fig**). Mps1 is also upregulated in basal-a tumor cell lines (**S3 Fig**).

### Mps1 expression is critical to TNBC breast cancer cell viability

Mps1 has been reported to be highly-expressed in TNBC [10, 21]. To investigate the role of Mps1 on cellular viability, the depletion of Mps1 by silencing RNA was performed (**S4 and S5 Figs**). An inducible short-hairpin RNA targeting Mps1 engineered into the TNBC/basal-a breast cell line (HCC1806). Doxycyclin-induced Mps1 knockdown triggered a dose-dependent reduction in cell viability of the HCC1806 TNBC tumor cell line (identified by Tyrpan Blue staining) concomitant with the reduction of target protein (**S4 Fig)**. To characterize the differential effect on cell viability between a transformed tumor cell line (BT-549) and a premalignant one (MCF10A), long-term cell viability (7 days) were used (**S5 Fig)**. Exposure to Mps1 siRNA induced a transient protein down-regulation evident 24 hours post-transfection and a full recovery of protein levels by day 5–6 post-transfection. Under similar kinetics of protein knockdown, BT549 and MCF10A cell lines have differential viabilities suggesting a potential differential requirement for Mps1. These findings are consistent with related studies of Mps1 depletion [10].

### Mps1 inhibitors developed as potent, selective inhibitors of catalytic activity

Because Mps1 is correlated with basal-a breast cancer (dysregulated expression, critical role in cell viability), the development of more specific chemical biology reagents were created to characterize the roles of Mps1 catalytic activity. PF-3837, PF-7006 were created and characterized to be highly-selective Mps1 inhibitors (**S1 Fig)**. Both inhibitors have high affinity for Mps1 (PF-7006 *K*_i_ = 0.27 ± 0.06 nM; PF-3837 K_i_ = 0.33 ± 0.04 nM). Phosphorylation of the histone H3 Ser-10 (HH3-Ser_10_) was used as a marker of Mps1 enzymatic activity because it is mediated by Aurora-B, coincident with chromosomal condensation, and decreases upon transition to anaphase (PF-7006 and PF-3837 do not inhibit Aurora-B, **S1 Table**) [33]. Both molecules potently inhibit Mps1 in MDA-MB-468 tumor cells as monitored by modulation of the proximal biomarker pHH3-Ser_10_ (PF-7006 IC_50_ = 2.5 ± 1.0 nM, n = 5; PF-3837 IC_50_ = 5.5 ± 2.1 nM, n = 10). To establish kinase selectivity, the two inhibitors were profiled at 1000 nM with a panel of 90 unique protein kinases under less stringent conditions (*K*_m_ concentration of ATP) (**S1 Table**). Under test conditions, 80% inhibition of kinase activity would be achieved for an inhibitor that had a *K*_i_ value of 125 nM and 90% inhibition would be for *K*_i_ = 60 nM. Follow-up 10-point dose-response analyses were performed using a complementary assay format (Caliper mobility-shift assay) to confirm potency. PF-3837 inhibited five kinases greater than 80% (% inhibition at 1000 nM, IC_50_)—JNK1 (98%, 42 nM), JNK2 (96%, 28 nM) DYRK1 (92%, 81 nM), TAK1 (91%, 290 nM), NUAK1 (88%, 52 nM). PF-7006 inhibited five kinases but is more selective for Mps1—JNK1 (97%, 220 nM), JNK2 (91%, 89 nM), DYRK1 (89%, 310 nM), NUAK1 (84%, 160 nM), ERK7 (81%). PF-3837 is 85-fold selective for Mps1 relative to the most potent off-target kinase while PF-7006 is even more kinase selective for Mps1 (330-fold). With the two inhibitors established to be potent, selective Mps1 inhibitors biochemically, functional cellular effects were studied to provide further evidence for specificity.

### Mps1 kinase inhibitors abrogate the mitotic checkpoint in basal-a breast cancer cells

**Fig 1:**
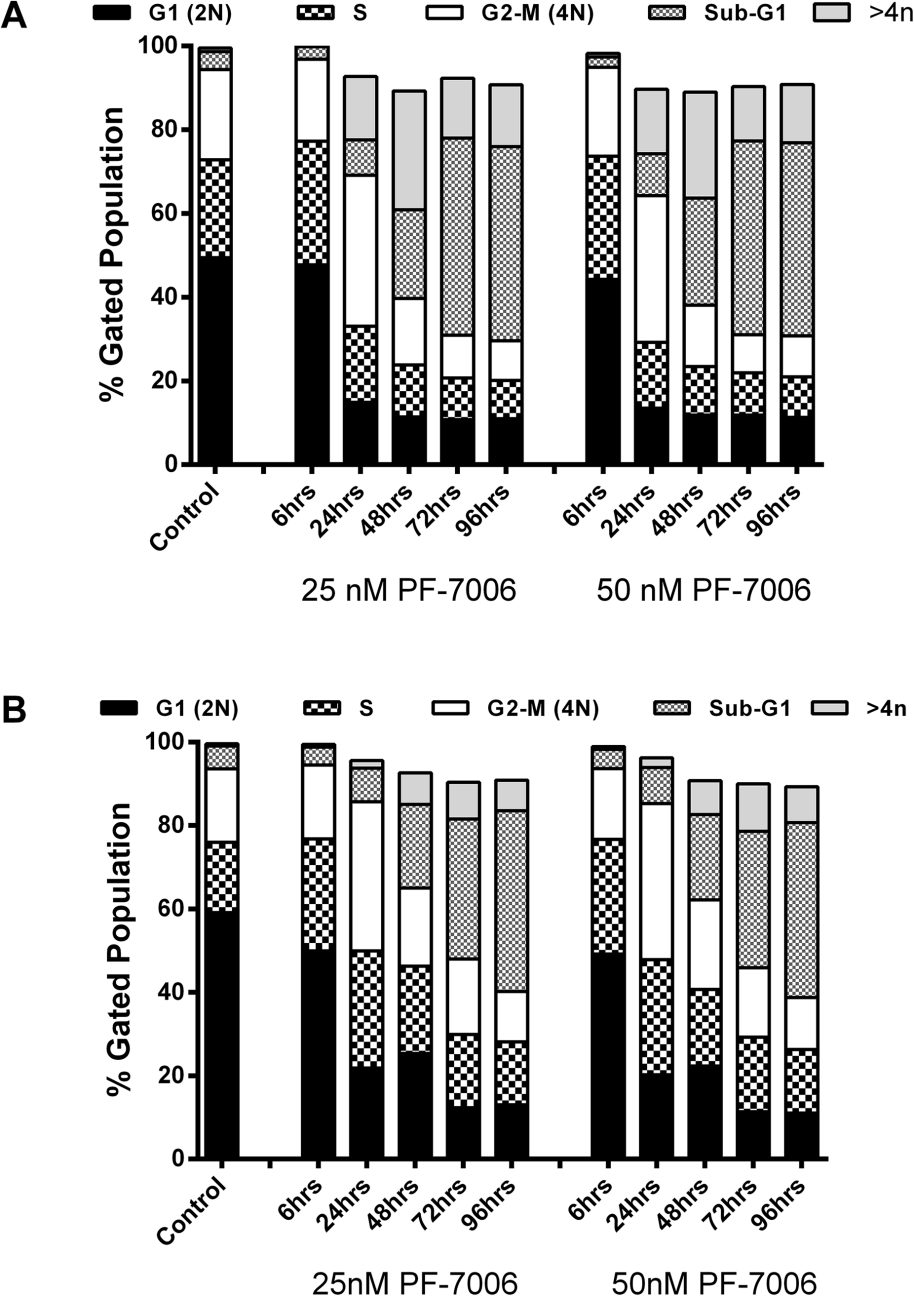
Analysis of cell cycle phenotypes induced by exposure of TNBC, basal-a tumor lines to selective Mps1 inhibitors. Flow cytometry analysis of the breast tumor lines HCC1806 (panel A) and HCC70 (panel B) exposed to one of two different concentrations of PF-7006 (25, 50 nM) for different treatment intervals in cells at various cell cycle stages. The result of analysis using propidium iodide (PI) staining is displayed.

**Fig 2:**
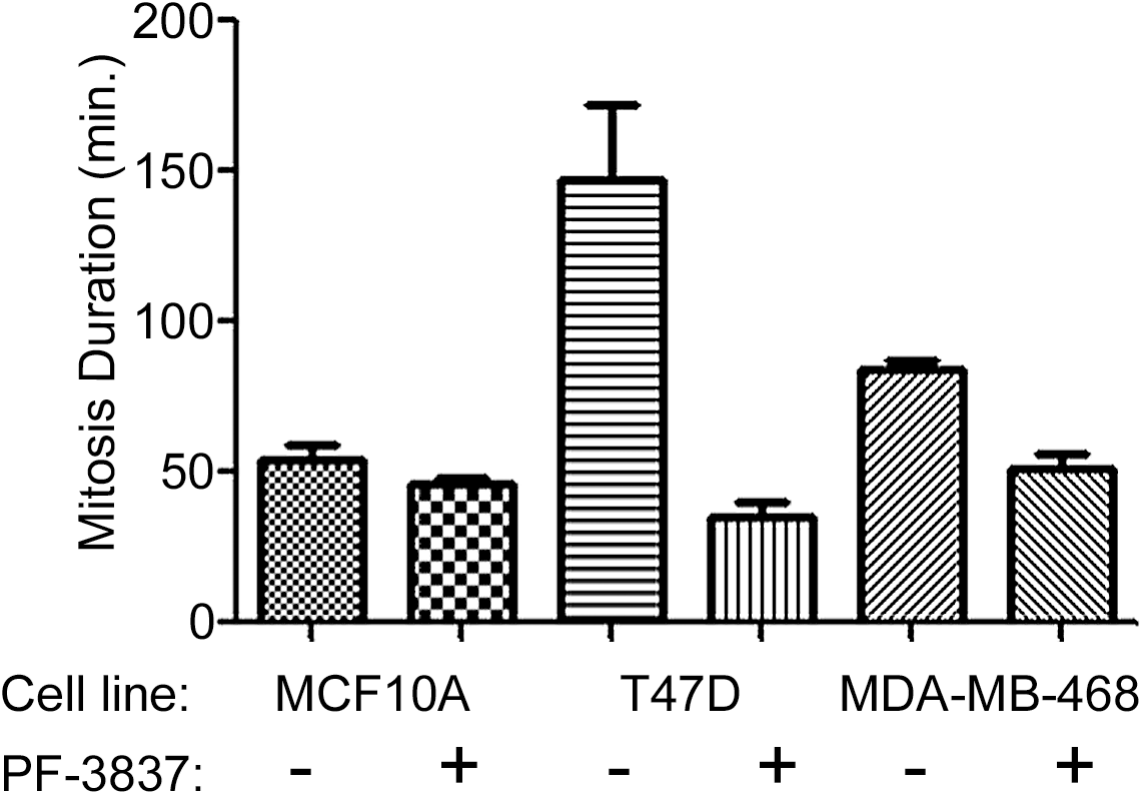
Effect of Mps1 inhibitor on duration of mitosis. Duration of mitosis timed from nuclear envelope breakdown to initiation of cytokinesis in three cell lines exposed to vehicle (0.1% DMSO) or an Mps1 inhibitor (50 nM PF-3837) using live-cell imaging (n = 3). The effect of PF-3837 on mitosis duration for tumor cells are highly-significant for T47D (p = 0.001) and MDA-MB-468 (p = 0.0002).

Phenotypic consequences of specific inhibition of Mps1 catalytic activity in a TNBC/basal-a breast cancer model (HCC1806 tumor cell line) were evaluated at multiple concentrations of PF-7006 as a function of time. To minimize the possible contribution of off-target effects, low concentrations of PF-7006 and PF-3837 were used. Flow cytometric analysis quantitated the cell cycle distribution with altered distributions (increased >4n DNA content and sub-G_1_ fractions) apparent after 24–48 hour treatment with a low concentration of PF-7006 (25 nM) (**Fig 1A, S6A Fig)**. Longer Mps1 inhibitor exposures (48–96 h) further increases the >4n DNA fraction. Similar trends are observed following exposure to 50 nM of PF-7006. Flow cytometric analysis in a second basal-a tumor cell line (HCC70) produced results similar to HCC1806 (**Fig 1B, S6B Fig)**. To visualize chromosome dynamics by fluorescent live-cell video microscopy, basal-a tumor cells (MDA-MB-468) were engineered to constitutively express the fluorescent reporter histone-2B green fluorescent protein (H2B-GFP). The acceleration of mitosis induced by Mps1 inhibition was quantitated using the Mps1 inhibitor PF-3837 in two tumor lines (T47D, MDA-MB-458) and one premalignant breast cell line (MCF10A) by measuring the time from nuclear envelope breakdown to the initiation of cytokinesis (**Fig 2**). Statistically significant decreases in the duration of mitosis was observed with low concentrations of PF-3837 (50 nM). Taken together, the inhibitors display mitotic functional properties consistent with Mps1 inhibition.

### Mps1 kinase inhibitors triggers signaling events consistent with mitotic checkpoint abrogation

**Fig 3:**
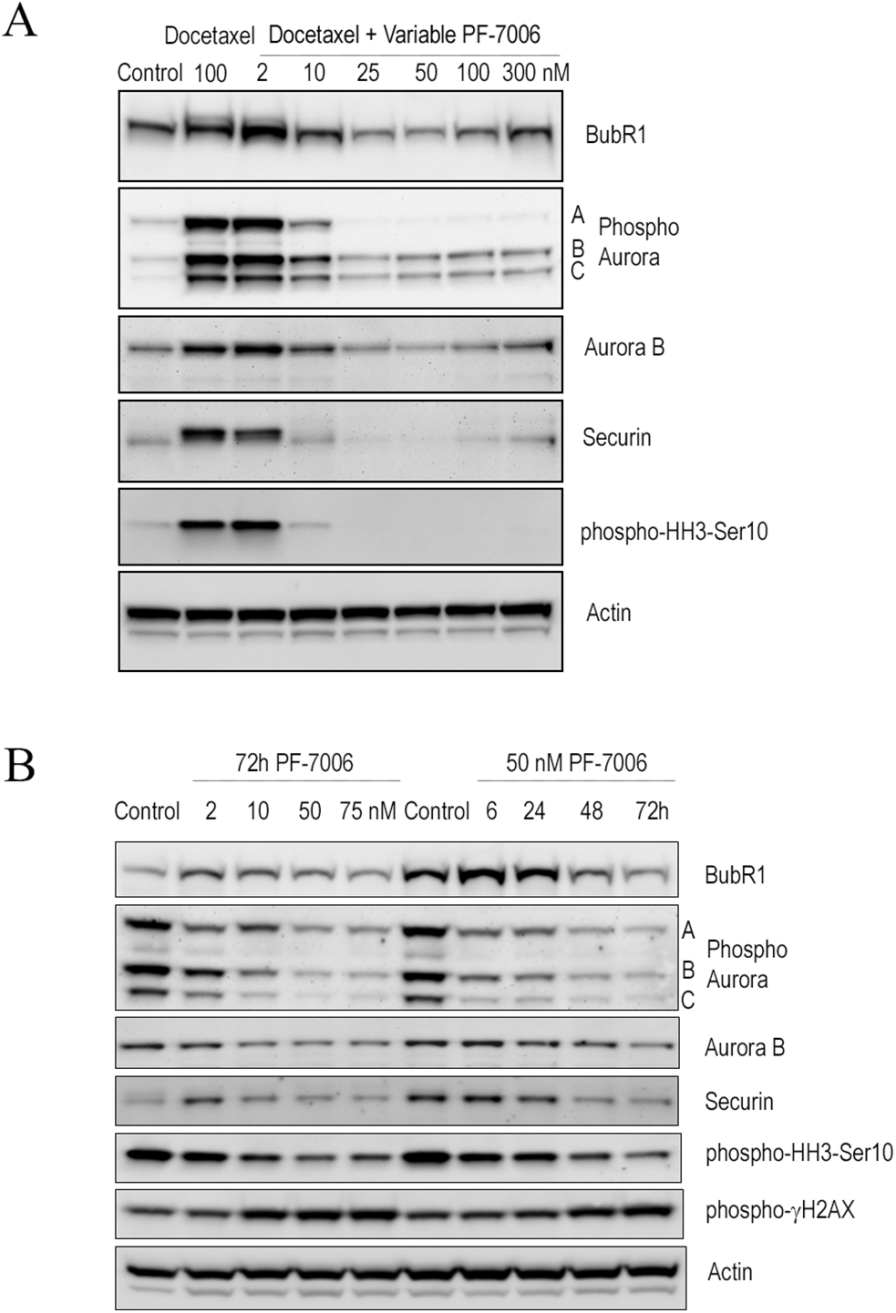
Impact on signaling events in response to PF-7006 as a function of time in synchronous and asynchronous cultures. (A) PF-7006-induced signaling effects on synchronous HCC1806 (docetaxel-treated) tumor cells. (B) PF-7006-induced signaling effects on asynchronous HCC1806 tumor cells.

To further explore the molecular underpinnings of inhibiting Mps1 catalytic activity, signaling proteins known to play critical roles in mitotic checkpoint activation were monitored after inhibitor treatment (**Fig 3**). Prior to exposure to an Mps1 inhibitor, cells were treated with docetaxel to activate the mitotic checkpoint and synchronize the cells. Microtubule stabilizing taxanes (docetaxel, paclitaxel) activate the mitotic checkpoint enriching an otherwise asynchronous cell population at the G2/M stage of the cell cycle [34]. Molecular indicators of a mitotic arrest such as pHH3-Ser_10_ are highest at the G_2_/M interphase and provide the pharmacodynamic approach to quantify checkpoint abrogation. Docetaxel (100 nM)-treated cells display the expected enhancement mitotic checkpoint activation proteins–BubR1, phospho-Aur-A/B/C, Aur-B, Securin and pHH3-Ser_10_ (**Fig 3A**). Upon subsequent treatment of PF-7006 (2–300 nM for 18 hours), the amounts of these checkpoint activation marker proteins or phosphoproteins detected decrease in a dose- and time-dependent manner consistent with checkpoint abrogation. As little as 10 nM of PF-7006 elicits approximately a 50% effect. To investigate whether the same pattern of changes in mitotic checkpoint proteins are observed in asynchronous cultures, HCC1806 cells were exposed to different concentrations of PF-7006 for 72 hours (**Fig 3B**). A trend for decreased presence of mitotic markers is apparent with Mps1 inhibitor exposure relative to the control cells (**Fig 3B**). The marker of double-stranded DNA breaks, γH2AX, shows the opposite trend, an increased numbers of positive cells concomitant with Mps1 inhibitor exposure. Again, 10 nM of PF-7006 for 72 hours was sufficient to double the levels of γH2AX relative to the control cells. To gain insight into the timing of the Mps1 inhibitor induced effects, HCC1806 cells were exposed to PF-7006 (50 nM) for various lengths of time. A decreased presence of mitotic checkpoint markers in the asynchronous HCC1806 tumor cells is evident as early as 6 hours of drug treatment and significantly decreased levels of these markers are apparent by 72h (**Fig 3B**). Similar to the observation with the taxane-synchronized cells, the levels of γH2AX have an enhanced abundance as a function of Mps1 inhibitor treatment time. Taken together, these molecular findings reveal mitotic effects consistent with selective inhibition of Mps1.

### Mps1 inhibition of catalytic activity evaluated in an orthotopic tumor model

**Fig 4:**
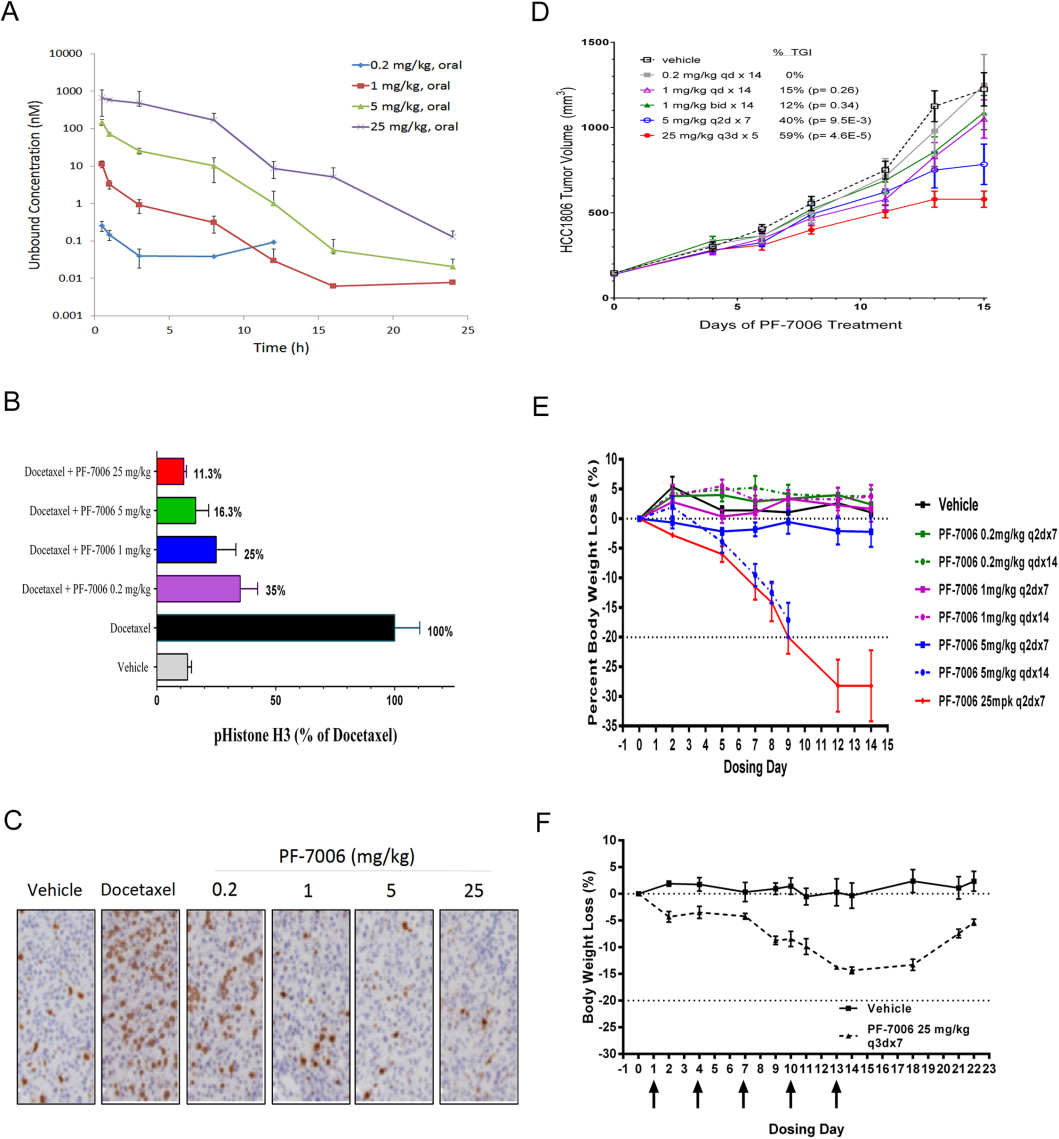
*In vivo* pharmacology of PF-7006 in the HCC1806 triple-negative breast cancer model. (A) Plasma concentration of PF-7006 following oral dosing of SCID mice (n = 3) to PF-7006 (0.2, 1, 5, 25 mg/kg). Error bars are standard deviations. (B) Modulation of mechanistic marker (pHH3-Ser_10_) in response to PF-7006 treatment as established by ELISA assay (n = 6). Error bars are standard deviations. (C) Modulation of pHH3-Ser_10_ in response to PF-7006 treatment based on tumor IHC (n = 3). (D) Tumor growth inhibition from PF-7006 treatment of HCC1806 tumor-bearing mice as a function of dosing and scheduling (n = 12). The following schedules were tested: 0.2 mg/k QD x 14 days (0% TGI), 1 mg/kg QD x 14 days (15% TGI, p = 0.26), 1 mg/kg BID x 14 (12% TGI, p = 0.34), 5 mg/kg Q2D x 7 (40% TGI, p = 9.5x10^-3^), and 25 mg/kg Q3D x 5 (59% TGI, p = 4.6x10^-5^). Error bars are standard error of the mean. (E) Effect of different doses and schedules of PF-7006 administration on body weights (n = 3). The following schedules were evaluated: 0.2 mg/kg Q2Dx7, 0.2 mg/kg Q2Dx14, 1 mg/kg Q2Dx7, 1 mg/kg QD x14, 5 mg/kg Q2Dx7, 5 mg/kg QD x 14 (significant body weight loss), and 25 mg/kg Q2Dx7 (significant body weight loss). Error bars are standard error of the mean. F. Effect on body weight based on intermittent PF-7006 dosing (25 mg/kg Q3Dx7, n = 3). Upon cessation of dosing, treated animals regained body weight. Error bars are standard error of the mean.

The Mps1 function was explored in orthotopic tumor models of basal-a breast cancer (HCC1806 cells implanted in SCID mice mammary fat pads). Free levels of drug in the plasma of tumor-bearing SCID mice were quantified following oral exposure to PF-7006 because, in theory, only unbound drug can engage a target (drug distribution is more complex than this simple model). Relatively rapid drug clearance was observed such that 25 mg/kg was necessary to sustain a plasma drug level (unbound) 3-fold above the pHH3-Ser_10_ cell-based EC_50_ (8 nM) for 10 hours (**Fig 4A**). Next we established two approaches to evaluate modulation of a mechanistic marker linked to mitotic checkpoint modulation, pHH3-Ser_10_ ELISA analysis and pHH3 immunohistochemistry (IHC). Dose-dependent modulation of pHH3-Ser_10_ in tumor explants from the orthotopic HCC1806 tumor model is evident following a 3 hour of drug exposure (**Fig 4B**) and at 0.5 and 8 hours (**S7 Fig)**. Docetaxel was used to engage the mitotic checkpoint which was quantitated with the pHH3-Ser_10_ biomarker. A PF-7006 dose-dependent decrease in pHH3-Ser_10_ levels was detected by both ELISA and IHC methodologies in tumor tissues (**Fig 4C**). These studies are consistent with PF-7006 as an Mps1 inhibitor. A tumor growth inhibition (TGI) study in the basal-a model of breast cancer HCC1806 implanted orthotopically in SCID mice resulted in a maximum TGI of 59% was observed upon oral administration of PF-7006 at 25 mg/kg every 3 days for 7 days (Q3Dx7) (**Fig 4D**) such that Mps1 was inhibited >90% periodically. More aggressive dosing schedules were not possible due to toleration issues (e.g. body weight loss).

### Mps1 inhibition affects normal physiology and limits toleration

**Fig 5:**
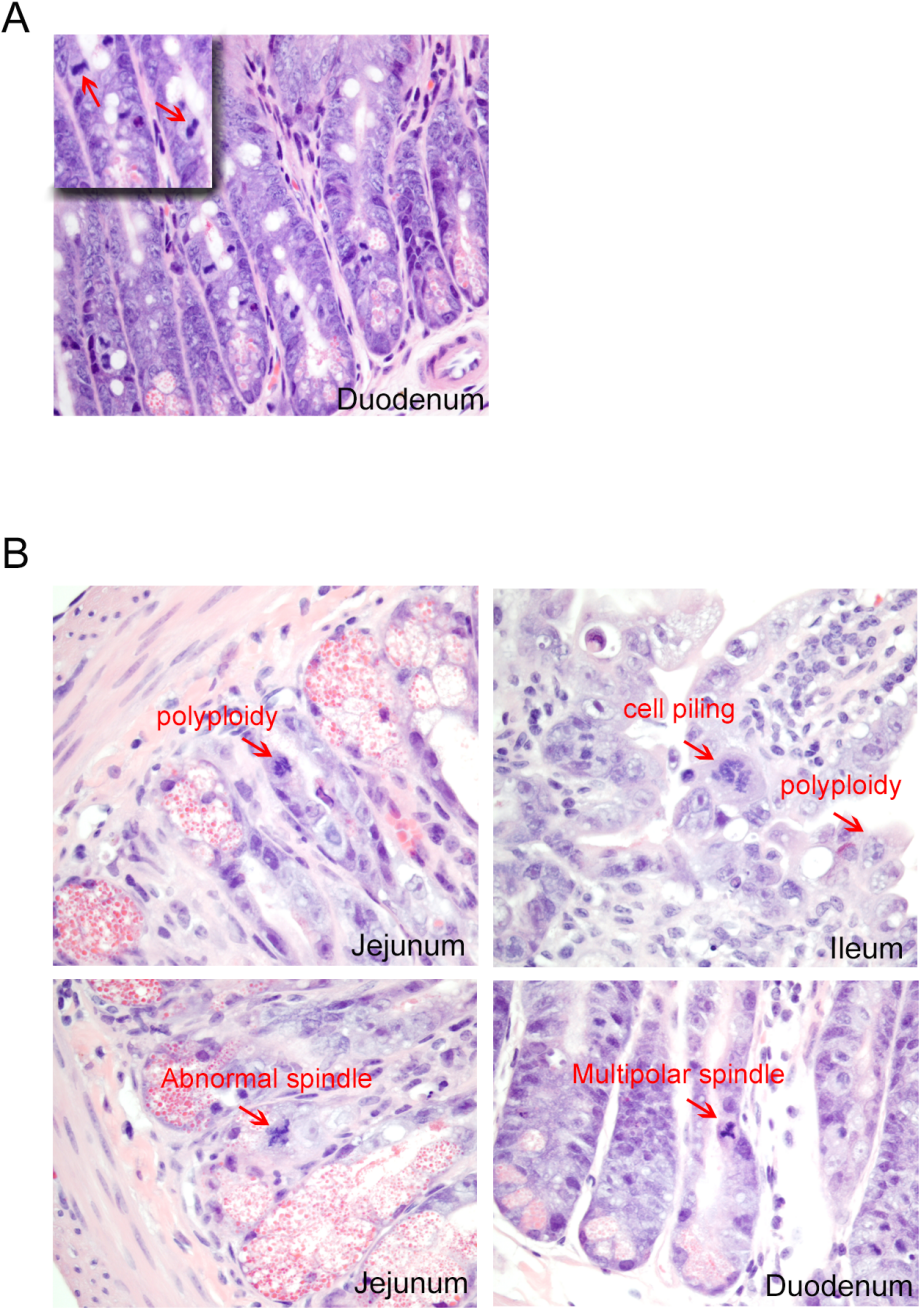
Histopathology of the small intestine of PF-7006-exposed mice. (A) Vehicle-treatment showing diploid mitotic Figs (arrows) (inset) magnification of the image (n = 3). (B) Representative images of histological segments of the small intestine (duodenum, jejunum, ileum) from PF-7006 treated mice (n = 3) displaying abnormal mitotic Figs (arrows).

Prolonged oral administration of an Mps1 inhibitor (PF-7006) to SCID mice triggered body weight loss that was dose–and schedule–dependent. Treatment schedules based on 25 mg/kg every other day for 7 days (q2dx7) or at 5 mg/kg daily for 14 days (qdx14) caused up to 20% body weight loss by Day 7 (**Fig 4E**). To explore the possibility that the dosing schedule could ameliorate toxicities, oral administration of 25 mg/kg of PF-7006 every three days for 2 weeks (q3dx5) resulted in a body weight loss of 15% by the end of dosing (**Fig 4F**). Body weight loss was reversible upon cessation of treatment. Compared to vehicle-treated animals, each region of the gastrointestinal tract (GI) displayed distinct mitotic aberrations including polyploidy and spindle abnormalities such as geometrical distortions and multi-polarity (**Fig 5B**). Intestinal epithelial cells were observed with polyploidy, abnormal spindles, multipolar spindles as well as cell piling (multilayer clusters in regions typically populated by a single layer). In contrast, mitoses observed in control tissues displayed normal bipolarity (**Fig 5A**) Bone marrow and spleen displayed marked reductions in hematopoietic cell density. Taken together, these findings are consistent with known Mps1-mediated biology and reveal a safety limitation to potently inhibiting Mps1 catalytic activity.

### Mps1 inhibitor-induced in vitro cytotoxicity mitigated with pretreatment of a Cdk4/Cdk6 inhibitor

**Fig 6:**
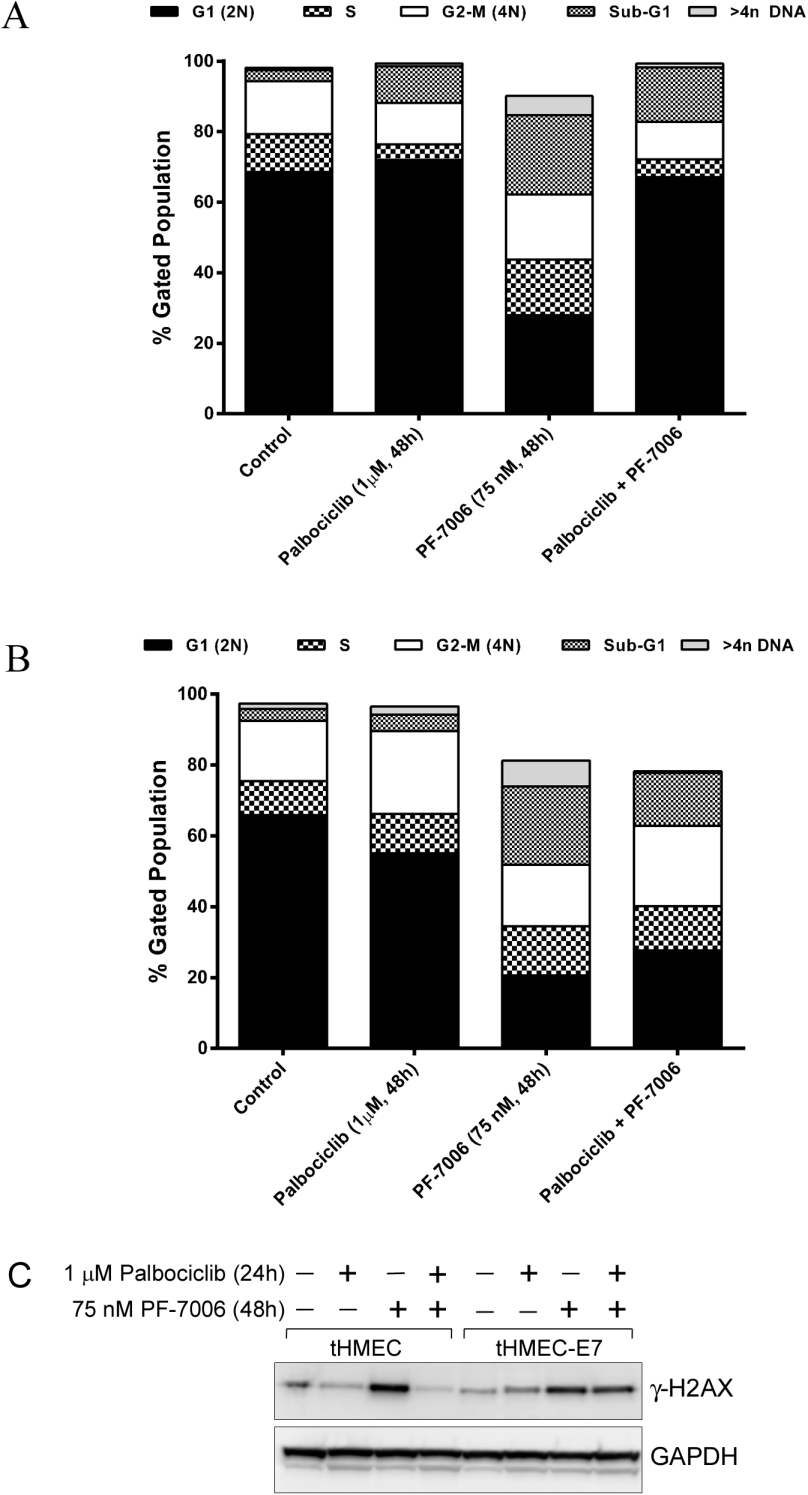
Mps1 PF-7006 inhibitor-induced cytotoxicity as function of retinoblastoma tumor suppressor protein (Rb1) status in tHMEC cultures co-treated with the CDK4/6 inhibitor palabociclib. Cells were treated with 1 μM palbociclib for 24 hours, 75 nM PF-7006 for 48 hours, or 24 hours of palbociclib (1 μM) followed by 48 hours of 75 nM PF-7006. (A) Flow cytometry analysis of Rb-competent tHMEC cells co-treated with Mps1 and CDK4/6 inhibitors. (B) Same experimental conditions as (A) applied to Rb-deficient tHMEC cells (tHMEC cells constitutively expressing the Human Papilloma Virus E7 oncogene). (C) Assessment of DNA damage (double-stranded breaks) based on γ-H2AX levels as a function of Rb status in tHMEC cells treated with CDK4/6 and Mps1 inhibitors.

To protect normal tissue from adverse effects of Mps1 inhibition, a chemoprevention strategy was explored. We hypothesized that inhibition of CDK4/6 would protect normal cells with an intact G_1_ restriction checkpoint (Rb1 competent) from Mps1 inhibitor-associated toxicities while remaining cytotoxic to tumor cells, such as basal-a breast cancer, which have lost the checkpoint. To test this hypothesis, an isogenic cell line model of Rb1 expression was created using a telomerase-immortalized human mammary epithelial line (tHMEC) engineered to express the human papilloma virus (HPV) E7 oncogene which inactivates Rb1 (**Fig 6**) [35, 36]. The isogenic pair of tHMEC cells (expression of vector alone or with E7 oncogene) was treated with different regimens of a CDK4/6 inhibitor (i.e. palbociclib) and a Mps1 inhibitor (e.g. PF-7006): 1 μM palbociclib for 48 hours, 75 nM PF-7006 for 48 hours, or palbociclib + PF-7006 (1 μM, 75 nM respectively). Cell cycle phenotypes were monitored by both immunofluorescence and by flow cytometry. Whereas the Rb1-positive cells displayed a normal cell cycle distribution (**Fig 6A**), the E7-expressing tHMEC Rb1-negative cells displayed a cell cycle profile enriched for 4n and 8n peaks as well as evidence of apoptotic cells (sub-G_1_ fraction) (**Fig 6B**). Immunofluorescence analysis revealed a pattern of nuclear staining that is consistent with the flow cytometry data (**S8 Fig)**. Rb1-competent cells displayed mono-nucleated morphology whereas the Rb1-negative cells displayed considerable micronucleation consistent with an attenuated mitotic checkpoint arising from an Mps1 functional block. Next, we tested the hypothesis that the CDK4/6 inhibitor prevents DNA damage induced by an Mps1 inhibitor in Rb1-negative but not in Rb1-positive cells. Pre-treatment for 48 hours with the CDK4/6 inhibitor followed by exposure to the Mps1 inhibitor PF-7006 induced dsDNA breaks in the Rb1-negative isogenic background but not in the Rb1-positive isogenic model as demonstrated by the level of expression of γ-H2AX, a marker of dsDNA breaks (**Fig 6C**). The tHMEC cells are protected by palbociclib (4^th^ column, upper band) because palbociclib reduces the γ-H2AX signal to background. The result is starkly different than in the Rb1 deficient cell line where palbociclib has no large effect on the induction of γ-H2AX (8^th^ column, upper band). The CDK4/6 chemoprevention strategy was also characterized in normal human bone marrow cells, an *in vitro* surrogate evaluation for the bone marrow hypocellularity observed in Mps1 inhibitor treated animals. Human bone marrow mononuclear cells were pretreated with palbociclib (100 and 300 nM) for 24 hours, followed by the treatment of Mps-1 inhibitor PF-7006 (100, 300, and 1000 nM). Caspase 3/7 activity was measured as a marker of apoptosis 24 hours after Mps-1 inhibitor treatment. Pretreatment with palbociclib rendered the human bone marrow mononuclear cells resistant to apoptosis induced by the PF-7006, as demonstrated by a robust decrease in caspase 3/7 activation in the bone marrow cells, compared to PF-7006 treatment alone (**S9 Fig)**. In addition, palbociclib pretreatment of IEC-6 gastrointestinal cells were also protected by palbociclib pretreatment (**S9 Fig)**. These cellular studies illustrate that there may be additional therapeutic uses for cytostatic cell cycle drugs.

## Discussion

The concept that chromosomal instability contributes to cancer dates back to 1902 from observations by Theodor Boveri [37]. Advances in cancer genomic sequencing support the theory that cancer progression proceeds by a mechanism of punctuated evolution enabled by CIN-generated genetic variants with strong selection pressure arising from the tumor microenvironment (i.e. hypoxia, chemotherapy) [12, 38]. Therefore, toleration to aneuploidy may have evolved to endow tumor cells with the genomic diversity required for their survival under selective pressure from the tumor microenvironment [39]. Central to this outcome are cell cycle processes that govern chromosomal integrity. To date, targeting the core proteins of the mitotic checkpoint has had mixed results [40]. A hypothesis tested in this work is whether tilting the balance of cancer cells towards excessive CIN will impart a tumor cytotoxic effect. Will speeding cells through mitosis be more effective than engaging the mitotic checkpoint and halting cell cycle progression?

Given the encouraging data from the Mps1 depletion studies, we sought to investigate Mps1 deeper using chemical biology approaches. Central to this analysis are small molecule inhibitors that block Mps1 catalytic activity. A caveat of all chemical biology studies is that there may be a contribution of off-target modulation of tumor biology which contributes to the observed pharmacology. The probability of this occurring increases with the dose of the inhibitor. This complication can be mitigated by studying multiple chemically-distinct inhibitors (off-target effects are usually compound-specific). Using high-throughput screening of a kinase-targeted compound library to identify promising chemical space, lead optimization (structure-based drug discovery) was used to create Mps1 inhibitors (PF-7006, PF-3837). Broad biological profiling of these Mps1 kinase inhibitors characterized them as potent and highly selective. Another measure of Mps1-selectivity is the ability of palbociclib to block cellular toxicities from the Mps1 inhibitors in Rb1-competent and not Rb1-deficient cells (isogenic pair). Cellular studies of these Mps1 inhibitors prove evidence of behaviors expected for a mechanism that induces mitotic checkpoint bypass: a) decrease in the presence of mitotic markers elevated upon taxane-induced mitotic arrest as well as the same markers in asynchronous cultures; b) elevated γ-H2AX; c) shorter mitoses; d) aneuploidy as confirmed by flow cytometry; and e) loss in cell viability and induction of apoptosis. Other Mps1 inhibitors have been reported to modulate the mitotic checkpoint in cellular studies with findings similar to those with PF-7006 and PF-3837 [17, 24, 26–29, 41]. For example, MPI-0479605 is an Mps1 inhibitor shown to induce mitotic checkpoint attenuation and induce aneuploidy *in vitro* [29]. NMS-P715 was characterized as selective Mps1 inhibitor (versus 60 kinase kinase panel) with a biochemical IC_50_ of 182 nM [22]. Cellular studies of NMS-P715 show mitotic checkpoint attenuation, acceleration of mitosis, aneuploidy, mitotic catastrophe and apoptosis [22]. From cellular studies, therapeutically targeting Mps1 seems to be a valid strategy which is consistent with previous proposals [21].

A strength of a chemical biological investigation of a potential therapeutic target is that it has expanded capacities relative to gene silencing analysis. We evaluated the role of selective Mps1 inhibition in tumor models. *In vivo* efficacy studies with PF-7006 using an orthotopic xenograft model of basal-a/TNBC breast cancer resulted in only 59% TGI at the maximum tolerated dose (MTD 25 mg/kg) using an intermittent dosing schedule (Q3x14). Pharmacodynamic studies show that each 25 mg/kg dose blocks ~85% of downstream Mps1 signaling. The modest tumor growth inhibition occurred at exposures that caused dose-limiting toxicities (e.g. body weight loss, neutropenia) likely to translate to the clinic. Both Mps1 inhibitors (PF-7006 and PF-3837) trigger similar mitotic aberrations both *in vitro* and *in vivo* consistent with an attenuated mitotic checkpoint resulting in chromosome mis-segregation and eventual apoptosis in rapidly dividing proliferative gastrointestinal compartments i.e. intestinal crypt and bone marrow. These findings reveal a critical role of Mps1 catalytic activity in normal physiology and therefore a potential limitation to therapeutically targeting it as a monotherapy. Similar efficacy and toleration findings have been reported but their impact on clinical utility was not illuminated. MPI-0479605 had modest efficacy in colorectal tumor xenograft models that ranged between TGI ranging from 50 to 70% [29]. Similar to our observations, MPI-0479605 dose-limiting toxicity was body weight loss and peripheral neutropenia [29]. *In vivo* studies of NMS-P715 also reveal modest efficacy (43–53% tumor growth inhibition) in xenograft models and no body weight loss [22]. Unfortunately, PK/PD analysis was not performed to confirm down-regulation of Mps1 activity nor was investigative pathology analysis reported. Further, the inhibitor used in the study is approximately 600-fold less potent than the inhibitors in the current study. Detailed studies of a potent Mps1 inhibitor 27f (imidazo[1,2-b]pyridazine) had modest efficacy (57% TGI) at a tolerated dose (2.5 mg/kg oral dosing) but caused significant body weight loss (25–32%) and death at higher doses (5, 10 mg/kg) [25]. The mechanism of toxicities was not investigated. Studies of MPS1-IN-3 inhibitor showed a modest survival advantage in tumor models without toxicities [28]. MPS1-IN-3 studies were done by twice weekly intravenous injections which affect the pharmacokinetic profile as well as minimize gastrointestinal toxicities. Studies on another chemical class of Mps1 inhibitor also resulted in only modest efficacy (66% TGI) [27]. The observed inhibitor toxicities in the current study are consistent with known Mps1 functional roles. In contrast to the Mps1 inhibitor studies, recent studies reveal that complete regression of basal-a/TNBC breast tumors in xenograft models can be achieved without significant body weight loss by targeting a different mitotic checkpoint protein that regulates another aspect of the checkpoint process (CENP-E) [31]. Taken together, Mps1 inhibitor mono-therapy, even with a highly proliferative tumor such as basal-a/TNBC, is likely to be limited by toxicities to normal proliferating cells.

To understand Mps1-direct toxicities, we sought ways to mitigate the adverse effects to normal physiology. CDK4/6 inhibitors are known to trigger cell cycle arrest in Rb1-competent cells [42–44]. Selective CDK4/6 inhibitors (e.g. palbociclib) are well-tolerated in the clinic with little gastrointestinal toxicity, uncomplicated neutropenia, leukopenia, anemia, and fatigue being the most common adverse events [45]. Because a common oncogenic defect in basal-a/TNBC tumor cells is the loss of the retinoblastoma tumor suppressor (Rb1) [46], a cytostatic CDK4/6 drugs that target Rb1 function was investigated as a mechanism to enhance the therapeutic window for Mps1 inhibition. The observations using an isogenic model for Rb1 expression (tHMEC-HPV-E16) provide evidence that in a combination regimen (palbociclib + PF-7006), cells would be spared from Mps1 inhibitor–induced genotoxicity in Rb1-competent but not in Rb1-incompetent cellular backgrounds. Normal bone marrow cells and gastrointestinal cells are protected from Mps1-mediated apoptosis by CDK4/6 inhibitor pretreatment. Others have reported similar findings for ionizing radiation and other anti-mitotic agents [47, 48]. These results support our hypothesis that Mps1-targeted therapy will be better tolerated when combined with CDK4/6 inhibition in Rb1-dysregulated cancers (e.g. HPV-induced cervical cancer, HPV+ head and neck squamous cell carcinomas). The combination (Mps1+CDK4/6) also may prove effective in a wider tumor segment in which the underlying oncogenic lesion is the loss of both Rb1 and p53 because these tumors are chromosomally unstable, aggressive, proliferative neoplasms. Further studies of the CDK4/6 drug palbociclib are merited to support its use in mitigating adverse events in patients treated with cytotoxic anti-mitotic therapies.

## Supporting Information

S1 Fig Chemical structures of Mps1 inhibitors PF-7006 and PF-3837. (TIF)

S2 Fig Mps1 expression in breast cancer is prognostic for disease-specific patient survival (480 patients, [32]). (TIF)

S3 Fig Mps1 expression analysis in breast cancer cell lines. (Top panel) Quantitation of Mps1 mRNA in tumor cells lines relative to GAPDH expression. Error bars are the standard deviation from samples tested in triplicate. (Bottom panel) Western blot analysis of Mps1 protein relative to a GAPDH protein control.
(TIF)

S4 Fig Impact on cell viability induced by genetic interference-mediated knockdown of Mps1 expression on the TNBC HCC1806 breast tumor cell line. Inducible shRNA-mediated depletion of Mps1 in the HCC1806 tumor line correlated with cell viability (n = 3). Error bars are the standard deviation.
(TIF)

S5 Fig Effect on cell viability induced by siRNA-induced Mps1 protein depletion. A. Effect on cell viability in the TNBC/Basal tumor line BT549 as a function of exposure to non-targeted or on-target siRNA (n = 3) (top) and assessment of protein knockdown using Western blot analysis (bottom). Error bars are the standard deviation. (B) Same analysis as Panel A but applied to the pre-malignant tumor line MCF10A. Error bars are the standard deviation.
(TIF)

S6 Fig Analysis of cell cycle phenotypes induced by exposure of TNBC, basal-a tumor lines to selective Mps1 inhibitors. FACS profiles of the breast tumor lines HCC1806 (panel A) and HCC70 (panel B) exposed to one of two different concentrations of PF-7006 (25 nM, 50 nM) for different treatment intervals in cells at various cell cycle stages using propidium iodide staining is displayed.
(TIF)

S7 Fig *In vivo* pharmacology of PF-7006 in the HCC1806 triple-negative breast cancer model (n = 6) with and without docetaxel treatment (25 mg/kg). Phospho-histone H3 (Ser_10_) was used as a pharmacodynamic marker. Error bars are the standard error of the mean.
(TIF)

S8 Fig Mps1 PF-7006 inhibitor-induced cytotoxicity as function of retinoblastoma tumor suppressor protein (Rb1) status in tHMEC cultures co-treated with the CDK4/6 inhibitor palabociclib. Cells were treated with 1 μM palbociclib for 24 hours, 75 nM PF-7006 for 48 hours, or 24 hours of palbociclib followed by 48 hours of PF-7006. The site of action of the Mps1 and CDK4/6 inhibitors is depicted to the left of this figure. (A) Immunofluorescence and flow cytometry analysis of Rb-competent tHMEC cells co-treated with Mps1 and CDK4/6 inhibitors. (B) Same experimental conditions as (A) applied to Rb-deficient tHMEC cells (tHMEC cells constitutively expressing the Human Papilloma Virus E7 oncogene).
(TIF)

S9 Fig *In vitro* evaluation of CDK4/6 inhibition as a chemoprevention strategy. (A) Human bone marrow cells were evaluated for the induction of apoptosis by measuring the activation of caspase-3 and -7 (n = 3). Error bars are the standard deviation. (B) IEC-6 rat gastrointestinal cells (small intestine epithelial cells) were evaluated for the induction of apoptosis by measuring the activation of caspase-3 and -7. For combination experiments (n = 3), IEC-6 cells were pre-incubated with 1 μM palbociclib for 24 hours at which time the media was removed and fresh media containing the combination of either DMSO or 1 μM palbociclib with the indicated doses of PF-3837 or PF-7006 was added for an additional 24 or 48 hours. Asterisks denote statistically significant differences between cells protected by 1 μM palbociclib relative to those without palbociclib treatment. Error bars are the standard deviation.
(TIF)

S1 Table Mps1 kinase inhibitors PF-7006 and PF-3837 were screened against panels of protein kinases in multiple screening formats. The International Centre for Kinase Profiling (Dundee) uses a radiometric screening assay and Invitrogen (Life Technologies) uses a fluorescence-based screening assay. Screening hits were followed up by Carna Biosciences using a mobility-shift assay format (Caliper Technologies). The results from these campaigns provide consistent findings.
(PDF)
