# Supplementary figures and images for: Mitotic Checkpoint Kinase Mps1 Has a Role in Normal Physiology which Impacts Clinical Utility
Source: PLoS One. 2015 Sep 23;10(9):e0138616. doi: 10.1371/journal.pone.0138616 (PMC4580473; doi:10.1371/journal.pone.0138616)

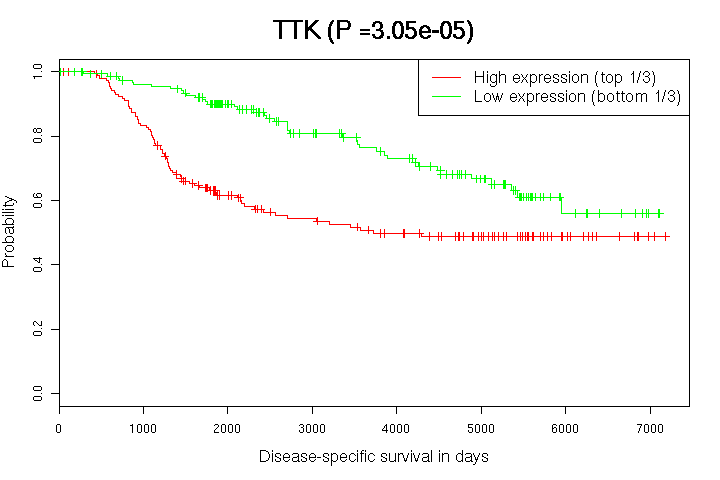

Supplement: S2 Fig — (TIF) [file pone.0138616.s002.tif]

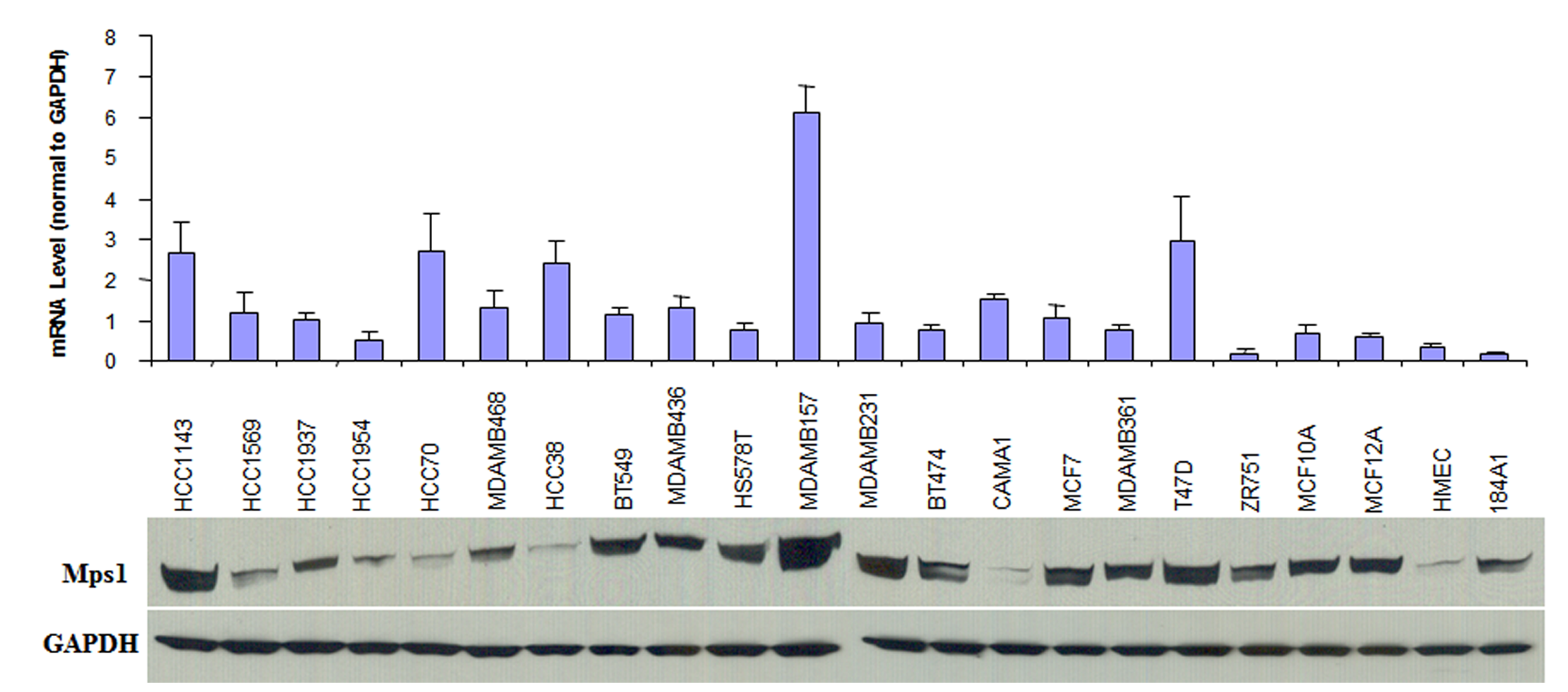

Supplement: S3 Fig — (Top panel) Quantitation of Mps1 mRNA in tumor cells lines relative to GAPDH expression. Error bars are the standard deviation from samples tested in triplicate. (Bottom panel) Western blot analysis of Mps1 protein relative to a GAPDH protein control. (TIF) [file pone.0138616.s003.tif]

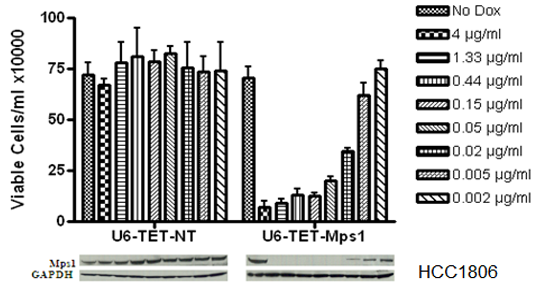

Supplement: S4 Fig — Inducible shRNA-mediated depletion of Mps1 in the HCC1806 tumor line correlated with cell viability (n = 3). Error bars are the standard deviation. (TIF) [file pone.0138616.s004.tif]

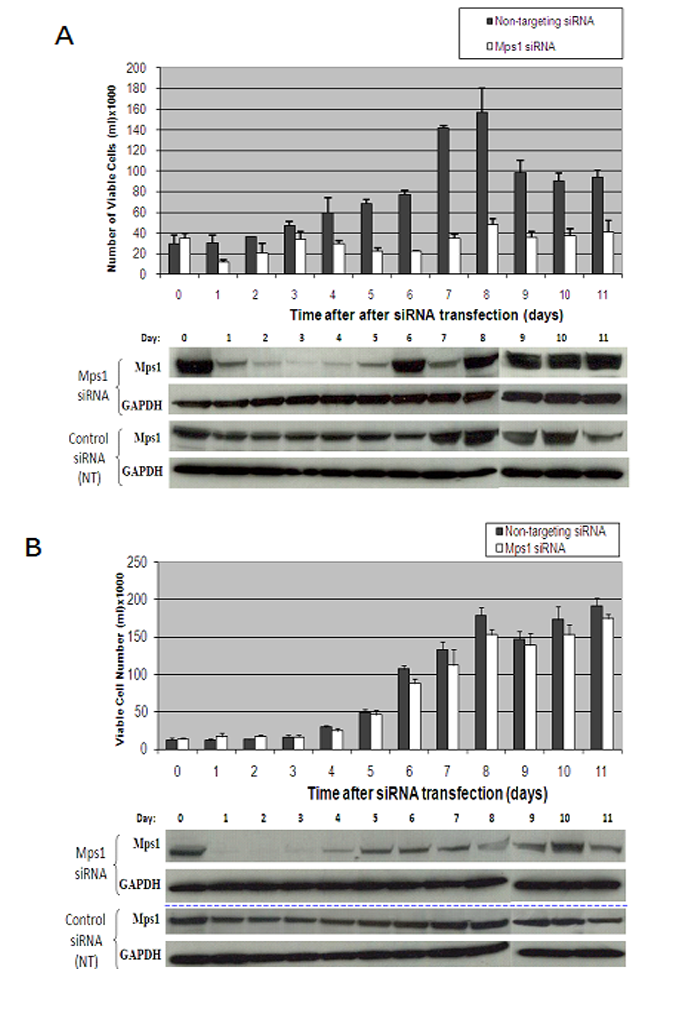

Supplement: S5 Fig — A. Effect on cell viability in the TNBC/Basal tumor line BT549 as a function of exposure to non-targeted or on-target siRNA (n = 3) (top) and assessment of protein knockdown using Western blot analysis (bottom). Error bars are the standard deviation. (B) Same analysis as Panel A but applied to the pre-malignant tumor line MCF10A. Error bars are the standard deviation. (TIF) [file pone.0138616.s005.tif]

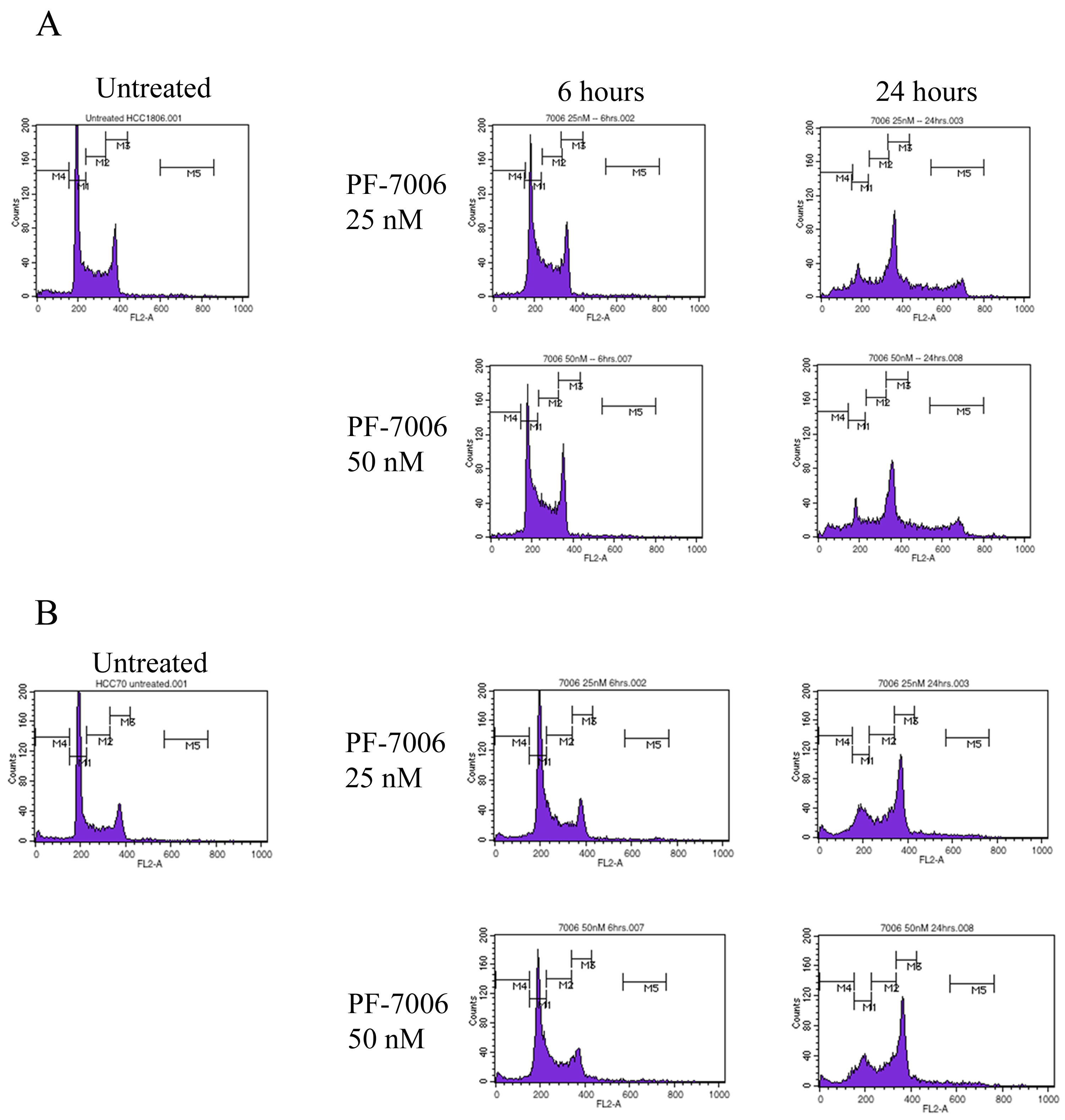

Supplement: S6 Fig — FACS profiles of the breast tumor lines HCC1806 (panel A) and HCC70 (panel B) exposed to one of two different concentrations of PF-7006 (25 nM, 50 nM) for different treatment intervals in cells at various cell cycle stages using propidium iodide staining is displayed. (TIF) [file pone.0138616.s006.tif]

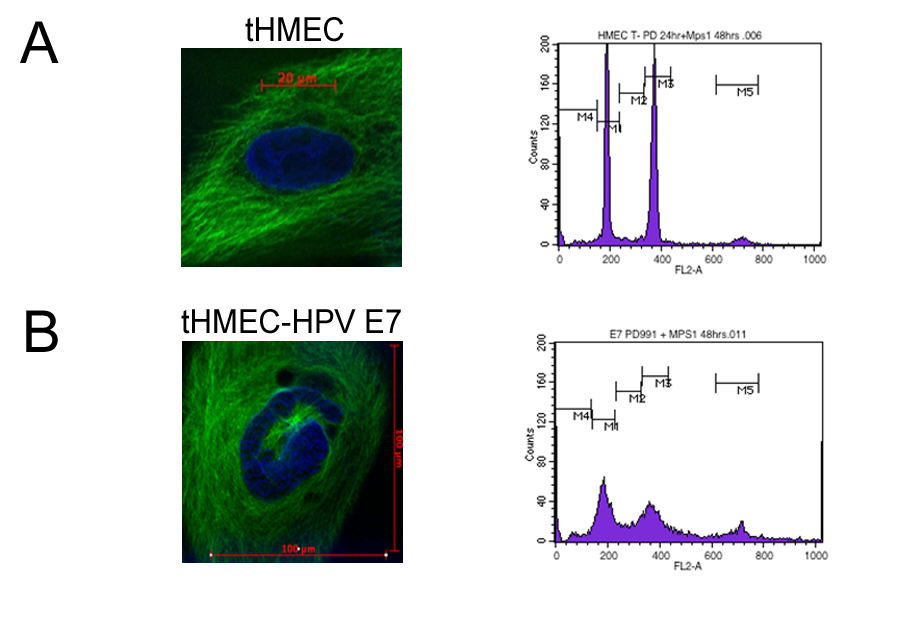

Supplement: S8 Fig — Cells were treated with 1 μM palbociclib for 24 hours, 75 nM PF-7006 for 48 hours, or 24 hours of palbociclib followed by 48 hours of PF-7006. The site of action of the Mps1 and CDK4/6 inhibitors is depicted to the left of this figure. (A) Immunofluorescence and flow cytometry analysis of Rb-competent tHMEC cells co-treated with Mps1 and CDK4/6 inhibitors. (B) Same experimental conditions as (A) applied to Rb-deficient tHMEC cells (tHMEC cells constitutively expressing the Human Papilloma Virus E7 oncogene). (TIF) [file pone.0138616.s008.tif]

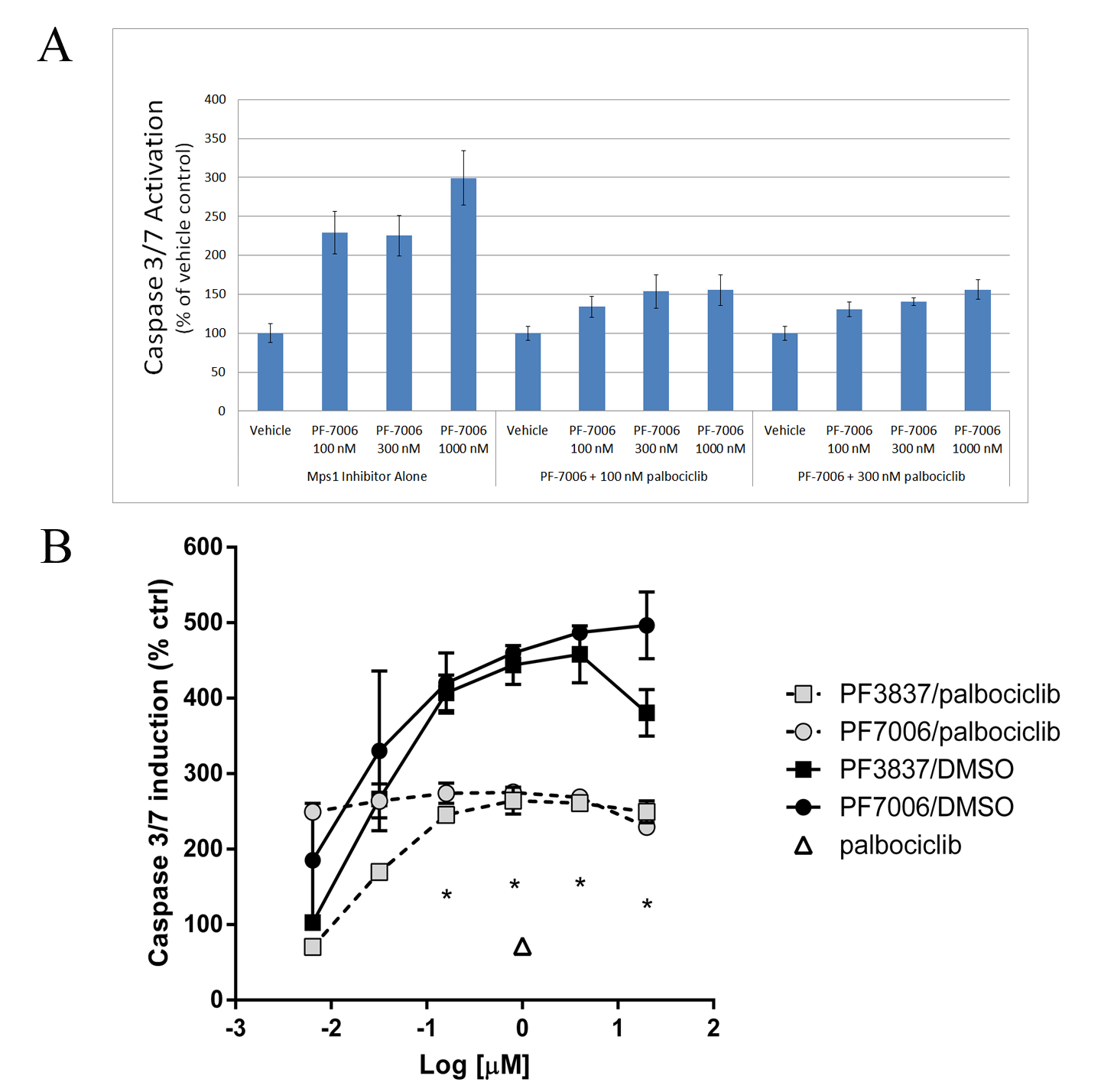

Supplement: S9 Fig — (A) Human bone marrow cells were evaluated for the induction of apoptosis by measuring the activation of caspase-3 and -7 (n = 3). Error bars are the standard deviation. (B) IEC-6 rat gastrointestinal cells (small intestine epithelial cells) were evaluated for the induction of apoptosis by measuring the activation of caspase-3 and -7. For combination experiments (n = 3), IEC-6 cells were pre-incubated with 1 μM palbociclib for 24 hours at which time the media was removed and fresh media containing the combination of either DMSO or 1 μM palbociclib with the indicated doses of PF-3837 or PF-7006 was added for an additional 24 or 48 hours. Asterisks denote statistically significant differences between cells protected by 1 μM palbociclib relative to those without palbociclib treatment. Error bars are the standard deviation. (TIF) [file pone.0138616.s009.tif]
