# Supplementary material for: Mitotic Checkpoint Kinase Mps1 Has a Role in Normal Physiology which Impacts Clinical Utility
Source: PLoS One. 2015 Sep 23;10(9):e0138616. doi: 10.1371/journal.pone.0138616 (PMC4580473; doi:10.1371/journal.pone.0138616)
Supplement: S1 Table — The International Centre for Kinase Profiling (Dundee) uses a radiometric screening assay and Invitrogen (Life Technologies) uses a fluorescence-based screening assay. Screening hits were followed up by Carna Biosciences using a mobility-shift assay format (Caliper Technologies). The results from these campaigns provide consistent findings. (PDF) [file pone.0138616.s010.pdf]

	PF-3837	PF-7006	
Kinase	% Inhibition at 1000 nM		Service
ABL1 (ABL)	11		Invitrogen
AKT1 (AKT)	-1		Dundee
AKT1 (AKT)	-2	-3	Invitrogen
AKT2	-7	-8	Dundee
AKT3	4		Invitrogen
AMPKa2 (rat PRKAA2)		10	Dundee
AURA (AURKA)	9	9	Invitrogen
AURB (AURKB)	19	17	Dundee
BTK	2		Invitrogen
CAMK1 (CaMK1a)	19	30	Dundee
CAMK2A (CaMKIIa)	11	4	Invitrogen
CAMKK2 (CAMKKB)	16	10	Dundee
CDK2 /CyclinA	65	39	Invitrogen
CHK1 (CHEK1)	6	6	Invitrogen
CHK2 (CHEK2)	41	15	Invitrogen
CKIa (CSNK1A1)	45	19	Invitrogen
CKId (rat CSNK1D)	45	79	Dundee
CKIIa (CSNK2A1)	19	27	Dundee
CKIIa (CSNK2A1)	10		Invitrogen
CKIIa (CSNK2A2 prime)	5	2	Invitrogen
CSK	-4	16	Dundee
DYRK1A (MNB)rat	92	89	Dundee
DYRK2	74	24	Dundee
EEF2K (EEF-2 kinase)	-6	7	Dundee
EGFR	8	0	Invitrogen
EphA2	3	-4	Invitrogen
ERBB4 (HER4)	34	13	Dundee
ERK2 (MAPK1)	32	62	Invitrogen
ERK7 (MAPK15)	79	81	Dundee
FGFR1	5	-1	Invitrogen
GCK (HK4)	31	28	Dundee
GSK3b	59	21	Invitrogen
IKKb (IKBKB)	21	14	Dundee
IRAK4	5	28	Dundee
IRK (INSR)	23	21	Invitrogen
JAK3	9	1	Invitrogen
JNK1a (MAPK8)	98	97	Dundee
JNK2 (MAPK9)	96	91	Dundee
KDR (KDRVEGF)	13	-4	Invitrogen
LCK	12	3	Invitrogen
LCK (murine)	6	20	Dundee
LKB1 (STK11)	20	26	Dundee
MAPKAPK2 (MK2)	6	2	Invitrogen
MAPKAPK5	4	-8	Dundee
MARK1	9	3	Invitrogen

Kinase
MLCK (MYLK)
MLCK_sk (MYLK2)
MLK1 (MAP3K9)
MLK3 (MAP3K11)
MNK1 (MKNK1)
MNK2 (MKNK2)
MSK1 (RPS6KA5)
MSK1 (RPS6KA5)
MSK2 (RPS6KA4)
MST2 STK3)
MST4
NEK2
NEK6
NTRK1 (TRKA)
NTRK1 (TRKA)
NuaK1
p38 (MAPK14) direct
p38d MAPK13)
p38g (MAPK12)
p70S6K (RPS6KB1)
PAK4
PBK
PDK1 (PDPK1) (directed)
PIM1
PIM2
PIM3
PKACa (PRKACA)
PKCa (PRKCA)
PKD1 (PRKD1)
PKG1 (PRKG1)
PKN1 (PRK1)
PKN2 (PRK2)
PLK1 preactivated
ROCK1 (ROCKI)
ROCK2 (ROCKII) rat
RSK1 (rat RPS6KA1)
RSK2 (RPS6KA3)
SGK1 (SGK)
SRC
SRPK1
TAK1 (MAP3K7)
TAOK2 (TAO2)
TIE2 (TEK)
TTK (Mps1)
ZAP70

MARK3	15	23	Dundee
MEK1 (rabbit MAP2K1)	26	66	Dundee
MET (HGFR)	-1	4	Invitrogen

ZC1 (MAP4K4)
ZC3 (MINK1)

PF-3837	PF-7006	
% Inhibition at 1000 nM		Service
18	35	Dundee
14	0	Invitrogen
0	16	Dundee
	35	Dundee
13	-14	Dundee
13	17	Dundee
8		Invitrogen
-4	11	Dundee
4		Invitrogen
23	2	Invitrogen
59	7	Invitrogen
-1	0	Invitrogen
8	16	Dundee
13	9	Dundee
11	25	Invitrogen
88	84	Dundee
1	5	Invitrogen
28	32	Dundee
3	23	Dundee
-3	-10	Dundee
18	-2	Invitrogen
44	36	Dundee
2	2	Invitrogen
9	23	Dundee
6	-5	Invitrogen
35	22	Dundee
1	-3	Invitrogen
-2	7	Dundee
71	57	Dundee
11		Invitrogen
5		Invitrogen
-9	23	Dundee
-2	-1	Dundee
6	2	Invitrogen
-3	22	Dundee
14	33	Dundee
-7	3	Dundee
8	3	Invitrogen
10	-2	Invitrogen
-1	10	Dundee
91	71	Dundee
23	3	Invitrogen
5	-1	Invitrogen
45	35	Dundee
6		Invitrogen

70	18	Invitrogen
40	17	Dundee
